# Comparing telemedicine and in-person gastrointestinal cancer genetic appointment outcomes during the COVID-19 pandemic

**DOI:** 10.1186/s13053-023-00250-8

**Published:** 2023-05-08

**Authors:** Samantha Williams, Jessica E. Ebrahimzadeh, Daniel Clay, Gillian Constantino, Jordan Heiman, Kirk J. Wangensteen, Kathleen Valverde, Nadim Mahmud, Bryson W. Katona

**Affiliations:** 1 Master of Science in Genetic Counseling Program, University of Pennsylvania Perelman School of Medicine, Philadelphia, PA USA; 2 Division of Hematology/Oncology, Department of Medicine, University of Pennsylvania Perelman School of Medicine, Philadelphia, PA USA; 3 Division of Gastroenterology and Hepatology, University of Pennsylvania Perelman School of Medicine, Philadelphia, PA USA; 4 Perelman Center for Advanced Medicine, University of Pennsylvania Perelman School of Medicine, 3400 Civic Center Blvd 751 South Pavilion, Philadelphia, PA 19104 USA

**Keywords:** Gastrointestinal cancer, Genetics services, Risk assessment, Telegenetics, COVID-19

## Abstract

**Background:**

The study purpose is to compare outcomes associated with completion of genetic testing between telemedicine and in-person gastrointestinal cancer risk assessment appointments during the COVID-19 pandemic.

**Methods:**

Data was collected on patients with scheduled appointments between July 2020 and June 2021 in a gastrointestinal cancer risk evaluation program (GI-CREP) that utilized both telemedicine and in-person visits throughout the COVID-19 pandemic, and a survey was administered.

**Results:**

A total of 293 patients had a GI-CREP appointment scheduled and completion rates of in-person versus telemedicine appointments were similar. Individuals diagnosed with cancer and those with Medicaid insurance had lower rates of appointment completion. Although telehealth was the preferred visit modality, there were no differences in recommending genetic testing nor in the consent rate for genetic testing between in-person and telemedicine visits. However, of patients who consented for genetic testing, more than three times more patients seen via telemedicine did not complete genetic testing compared to those seen in-person (18.3% versus 5.2%, p = 0.008). Furthermore, telemedicine visits had a longer turnaround time for genetic test reporting (32 days versus 13 days, p < 0.001).

**Conclusions:**

Compared to in-person GI-CREP appointments, telemedicine was associated with lower rates of genetic testing completion, and longer turnaround time for results.

**Supplementary Information:**

The online version contains supplementary material available at 10.1186/s13053-023-00250-8.

## Background

Germline variants in cancer-associated genes can be detected in 5–10% of individuals with cancer [1–3]. Identification of these inherited cancer risk syndromes has been facilitated by the development of cancer risk evaluation programs, which can often be specialized, such as those focusing on hereditary gastrointestinal (GI) cancer syndromes [4]. Identifying individuals with hereditary GI cancer risk syndromes is critical to ensuring that appropriate cancer risk management strategies are implemented to prevent development of cancer or detect cancers at an earlier stage [5]. Further, identification of hereditary cancer risk extends beyond the individual by allowing cascade testing of at-risk relatives [6].

Despite its importance, barriers to GI cancer risk evaluation exist. A barrier to healthcare, defined as anything that restricts or makes it more difficult for a patient to access care, can include transportation access and costs, health literacy, lack of health insurance, cost of medical care, and language barriers among others [7]. In addition to barriers, racial and ethnic disparities in care have been identified in medical oncology [8–10], and there is a growing need to better study these disparities in cancer-related genetic counseling. A prior study by our group focusing on appointment completion rate in a GI cancer risk evaluation program (GI-CREP) found that Medicaid insurance coverage, self-reported Black race, and a personal history of cancer were all associated with lower rates of GI cancer risk evaluation appointment completion [11].

The COVID-19 pandemic has led to substantial changes in healthcare delivery, including increased utilization of telemedicine; however, how these changes affect the delivery of cancer risk assessment services remains largely uncharacterized. In 2019, prior to the start of the pandemic, only 8% of Americans had seen a healthcare provider via telemedicine, whereas during the COVID-19 pandemic, a rapid increase in telemedicine utilization was observed [12]. Telemedicine can improve access to care by reducing both temporal and monetary costs associated with travel [13]. Despite the well documented benefits of telemedicine there are also specific barriers to its use. Telemedicine may be infeasible for patients without reliable access to a computer, smartphone, or the internet. Furthermore, technological literacy may also be a significant barrier and is among the most common challenges reported by older adults [14].

One of the largest systemic barriers to widespread telemedicine access is insurance coverage and reimbursement methods for providers. The 2018 Bipartisan Budget Act was a step towards increased telemedicine access for Medicare beneficiaries, as it removed the minimum distance requirement between patient and provider for telemedicine reimbursement [15]. Another potential barrier to cancer risk evaluation via telemedicine is the uptake and successful completion of genetic testing. Apart from potential differences in patient consent to undergo genetic testing, those seen via telemedicine must coordinate sample collection after their visit is completed; this involves arranging a blood draw at a designated site or independently collecting a saliva sample at home, which increases the potential for delays or errors, such as incorrect labeling of specimens.

Understanding differences in outcomes between in-person and telemedicine use of cancer risk evaluation services is critical given the likely continued use of telemedicine as a frequently utilized healthcare delivery model moving forward. In particular, it is essential to understand and compare appointment and genetic testing outcomes for both in-person and telemedicine visits. Given that the GI-CREP at our institution maintained both telemedicine and in-person appointments throughout the COVID-19 pandemic, this unique clinic structure allowed comparison of cancer risk evaluation outcomes between these two groups.

## Methods

### Study design, selection criteria, and data collection

This was a two-part study comprised of a retrospective cohort study and qualitative patient survey, with all study related activities being approved by the University of Pennsylvania Institutional Review Board. For the retrospective cohort study, the University of Pennsylvania Institutional Review Board granted a waiver of informed consent. Medical records were reviewed for all patients scheduled in the Penn Medicine GI-CREP clinic between July 2020 and June 2021, including patients who completed a GI-CREP appointment as well as patients who were scheduled but did not complete a GI-CREP appointment (either due to not attending a scheduled appointment or cancelling a scheduled appointment without rescheduling). Patients with previous cancer-focused genetic testing or who were not being seen for cancer risk evaluation were excluded. At the time of scheduling, patients were offered the soonest available appointment regardless of whether the visit was in-person or telemedicine, and if desired, patients could wait for their preferred visit type. During the study period, wait times for telemedicine appointments (generally 4–6 months) were typically twice as long as wait times for in-person appointments (generally 2–3 months). All GI-CREP appointments included an evaluation by both a genetic counselor as well as a gastroenterologist with cancer genetics expertise, and the recommendation for patients to undergo genetic testing was typically based on guidelines from the National Comprehensive Cancer Network (NCCN).

Patients seen for in-person appointments who agreed to undergo genetic testing had the option of providing a saliva sample in clinic or providing a blood sample from a lab within the same building as the GI-CREP clinic. Patients seen via telemedicine who consented to genetic testing could provide a sample through a saliva kit that would be mailed to their residence or blood collection arranged either remotely or onsite at the clinic at a later date. Orders placed by providers for genetic testing were typically valid for 1 year, and at the time of this study, the GI-CREP clinic had no formal process in place to address samples that were not returned within a set amount of time. All relevant data were collected from the electronic medical record, including demographics (age, sex, race), insurance coverage, marital status, religion, personal history of cancer, reason for referral to genetics clinic, whether genetic testing was recommended during the patient visit, patient consent to genetic testing, and patient completion of genetic testing. Distance to center was calculated as the shortest driving distance in miles between the patient’s home address and hospital address. Median income was calculated using the U.S. Census Bureau’s 2016–2020 American Community Survey 5-Year Estimates (United States Census Bureau, 2020) using the zip code of the patient’s residence.

A qualitative survey using a 7-question opinion-based survey was administered verbally to patients in the GI-CREP clinic at the end of their appointment. Participation in this survey was offered to all patients seen in GI-CREP via in-person appointment or telemedicine between March 2021-June 2021, with 66 patients agreeing to participate in the survey. The administering healthcare provider obtained verbal consent prior to survey administration, and acknowledgement of consent was documented in the patient’s chart along with their survey responses.

### Statistical analysis

Descriptive statistics were reported as medians and interquartile ranges for continuous data and as frequencies and percentages for categorical data. Comparisons were made as stratified by appointment completion status using the Wilcoxon rank-sum and chi-square tests for continuous and categorical data, respectively. Among patients who completed a GI-CREP appointment, we compared in-person and telemedicine visits using similar methods.

To identify variables associated with appointment completion, we used a logistic regression approach. We first evaluated all potential covariates in univariable models and retained variables with p < 0.10 for evaluation in subsequent multivariable models. The final model retained variables with p < 0.05. To visualize the results of this model, we computed marginal predicted probabilities and plotted these as a function of reason for referral and insurance status. We used a similar methodological approach to fit a model for the outcome of successful completion of genetic testing, which was evaluated in the subcohort of patients where genetic testing was recommended. To identify potential differences in time to complete genetic testing (in days) between in-person and telemedicine visits, we plotted kernel density functions and compared distribution medians using the Wilcoxon rank-sum test. Finally, results from the qualitative patient surveys were presented descriptively with comparisons between in-person and telemedicine visits, and specific reasons for preferring one visit modality or another were also presented. All data analysis was performed using STATA 17.0/BE (College Station, TX).

## Results

### Cohort characteristics

**Fig. 1:**
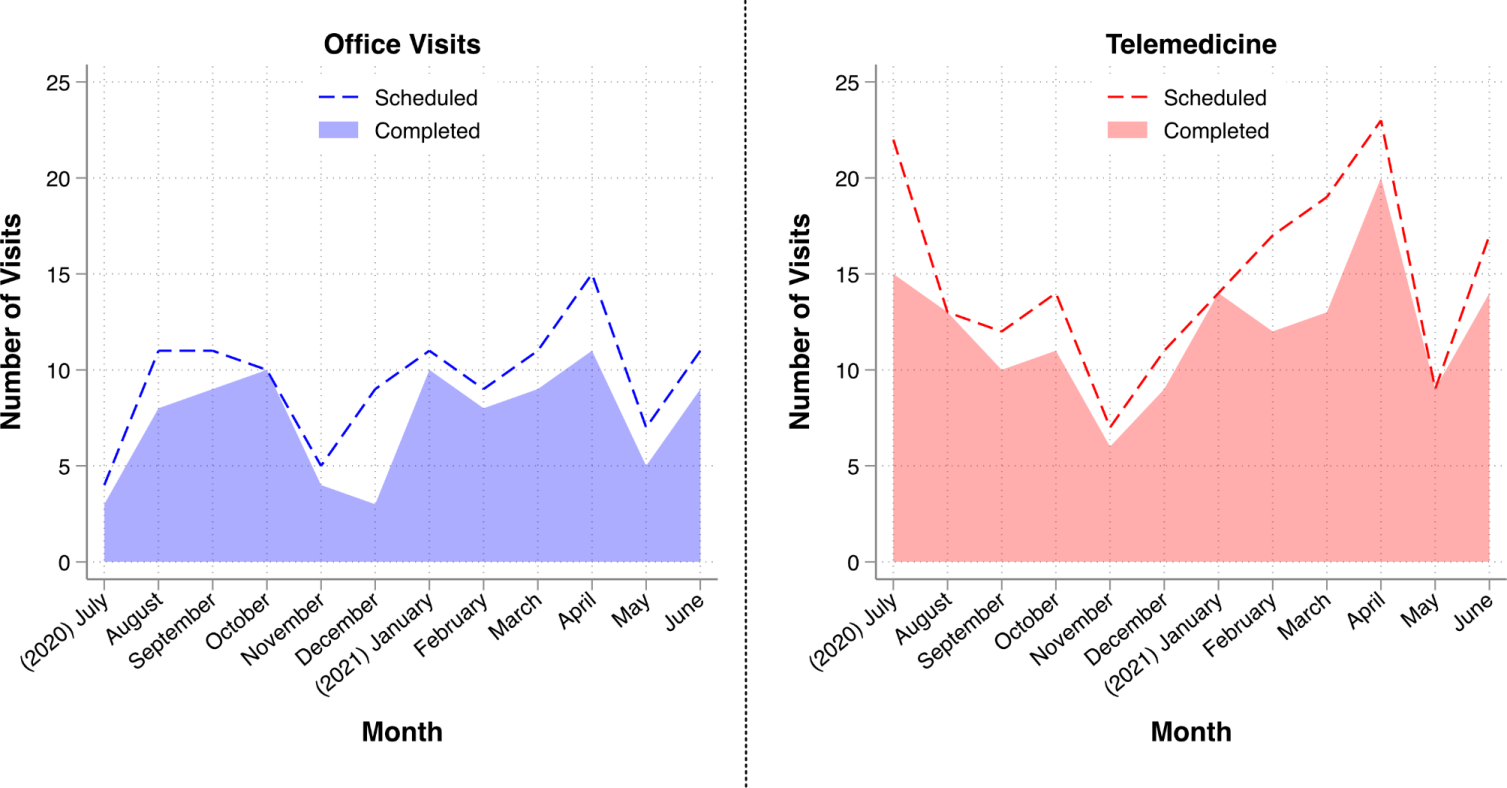
Scheduled and completed GI-CREP appointments by visit type. Number of scheduled and completed in-person office visits and telemedicine visits are plotted by month over the duration of the study period from July 2020 through June 2021

Of 293 patients scheduled in GI-CREP between July 2020-June 2021, 179 (61%) were scheduled for a telemedicine appointment, while 114 (39%) were scheduled for an in-person appointment (Table 1). A GI-CREP appointment was completed by 236 (81%) patients, whereas an appointment was not completed by 57 (19%) (Table 1). The in-person office visits and telemedicine visits were relatively uniformly distributed over the course of the study period from July 2020-June 2021, with telemedicine visits more common overall (Fig. 1). This cohort consisted primarily of individuals who were female, white, married, with private health insurance, and who were referred to GI-CREP for a personal or family history of cancer (Table 1).

**Table 1:** Cohort characteristics by appointment completion status and modality amongst patients scheduled for a GI-CREP appointment

	Appointment Completion (N = 293)			Type of Appointment Completed (N = 236)		
Factor	Appointment Completed (N = 236)	Appointment Not Completed (N = 57)	p value	In-Person Office Visit (N = 89)	Telemedicine (N = 147)	p value
**Age, median (IQR)**	49 (37, 60.5)	54 (44, 65)	0.064	53 (38, 65)	45 (36, 57)	0.013
**Sex**						
Female	129 (54.7%)	35 (61.4%)	0.36	50 (56.2%)	79 (53.7%)	0.72
Male	107 (45.3%)	22 (38.6%)		39 (43.8%)	68 (46.3%)	
**Race**						
White	174 (73.7%)	38 (66.7%)	0.22	65 (73.0%)	109 (74.1%)	0.75
Black	28 (11.9%)	8 (14.0%)		9 (10.1%)	19 (12.9%)	
Hispanic	9 (3.8%)	0 (0.0%)		5 (5.6%)	4 (2.7%)	
Asian	11 (4.7%)	4 (7.0%)		5 (5.6%)	6 (4.1%)	
Other	14 (5.9%)	7 (12.3%)		5 (5.6%)	9 (6.1%)	
**Marital Status**						
Single	83 (35.2%)	22 (38.6%)	0.63	29 (32.6%)	54 (36.7%)	0.52
Married	153 (64.8%)	35 (61.4%)		60 (67.4%)	93 (63.3%)	
**Religion**						
Christian	115 (48.7%)	27 (47.4%)	0.25	45 (50.6%)	70 (47.6%)	0.24
Jewish	29 (12.3%)	5 (8.8%)		14 (15.7%)	15 (10.2%)	
Muslim	5 (2.1%)	4 (7.0%)		3 (3.4%)	2 (1.4%)	
Other	87 (36.9%)	21 (36.8%)		27 (30.3%)	60 (40.8%)	
**Insurance**						
Private	173 (73.3%)	29 (50.9%)	< 0.001	60 (67.4%)	113 (76.9%)	0.19
Medicare	45 (19.1%)	15 (26.3%)		22 (24.7%)	23 (15.6%)	
Medicaid	17 (7.2%)	9 (15.8%)		6 (6.7%)	11 (7.5%)	
Other/Unknown	1 (0.4%)	4 (7.0%)		1 (1.1%)	0 (0.0%)	
**Distance to Center, median (IQR)**	19.75 (8.8, 33.95)	21.4 (11.9, 32.7)	0.30	16.3 (7.1, 33.5)	20.4 (9.6, 34.3)	0.50
**Median Income ($), median (IQR)**	82571.5 (66230.5, 106,778)	93,571 (69,305, 104,928)	0.51	81,519 (64,237, 104,318)	84,632 (68,224, 107,388)	0.72
**Personal History of Cancer**						
No	147 (62.3%)	15 (30.0%)	< 0.001	62 (69.7%)	85 (57.8%)	0.069
Yes	89 (37.7%)	35 (70.0%)		27 (30.3%)	62 (42.2%)	
**Referral Reason**						
Personal history of cancer	80 (33.9%)	34 (59.6%)	< 0.001	22 (24.7%)	58 (39.5%)	0.24
Family history of cancer	85 (36.0%)	10 (17.5%)		36 (40.4%)	49 (33.3%)	
Personal history of polyps	47 (19.9%)	2 (3.5%)		21 (23.6%)	26 (17.7%)	
Family history of genetic syndrome	17 (7.2%)	2 (3.5%)		7 (7.9%)	10 (6.8%)	
Other	7 (3.0%)	9 (15.8%)		0 (0.0%)	0 (0.0%)	
**Visit Type**						
In-person	89 (37.7%)	25 (43.9%)	0.39			
Telemedicine	147 (62.3%)	32 (56.1%)				
**Genetic Testing Recommended**						
No				5 (5.6%)	11 (7.5%)	0.58
Yes				84 (94.4%)	136 (92.5%)	
**Consented for Genetic Testing** **(if recommended)**						
No				7 (8.3%)	10 (7.4%)	﻿0.79
Yes				77 (91.7%)	126 (92.6%)	
**Genetic Testing Completed** **(if consented)**						
No				4 (5.2%)	23 (18.3%)	0.008
Yes				73 (94.8%)	103 (81.7%)	

### Variables Associated with Appointment Completion

**Fig. 2:**
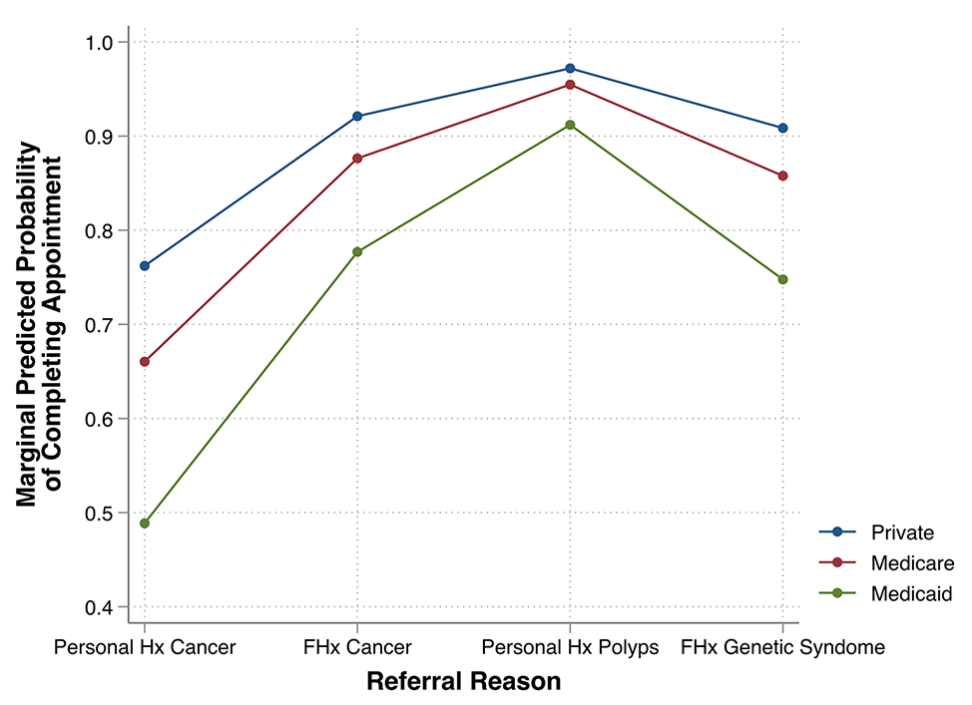
Predicted probabilities of appointment completion from multivariable logistic regression model. The marginal predicted probability of completing an appointment for individuals with private insurance, Medicare, and Medicaid is plotted by referral reason

Whether the GI-CREP appointment was in-person or telemedicine was not significantly associated with the rate of appointment completion (p = 0.39). Similarly, patient age, sex, race, marital status, religion, distance to the center, and median income were not significantly associated with appointment completion. In multivariable analyses, reason for referral and insurance status were both significantly associated with appointment completion. Specifically, referral for a personal history of cancer (OR 0.27, 95% CI 0.12–0.62, p = 0.002) was associated with significantly lower odds of appointment completion (Supplementary Table 1) relative to patients referred for a family history of cancer. While only 42.3% of the cohort had a personal history of cancer, this sub-group accounted for 70% of non-completed appointments. Patients with Medicaid insurance had a significantly lower odds of appointment completion relative to those with private insurance (OR 0.30, 95% CI 0.11–0.82, p = 0.02; Supplementary Table 1). These results are summarized in plots of marginal predicted probabilities of appointment completion, which was highest in privately-insured patients with a personal history of polyps and lowest in Medicaid patients with a personal history of cancer (Fig. 2).

### Variables Associated with Completion of genetic testing

**Fig. 3:**
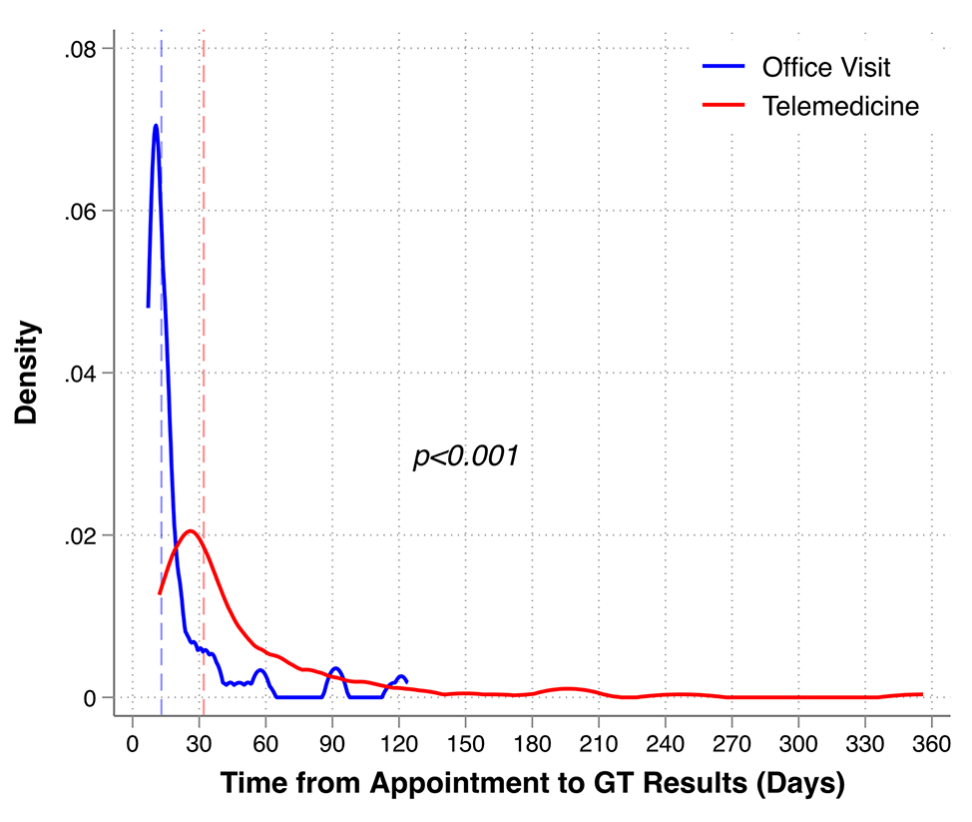
Distribution of time between GI-CREP appointment and return of genetic testing results. For patients completing genetic testing, the time between their appointment date and return of genetic testing (GT) results is plotted. Median times indicated by vertical dashed lines. Median time for in-person office visits is 13 days (IQR 9, 18), whereas median time for telemedicine visits is 32 days (IQR 22, 56). p < 0.001 by the Wilcoxon Rank-Sum test

Of the 236 patients that completed a GI-CREP appointment, 89 (38%) did so via an in-person office visit, whereas 147 (62%) did so via telemedicine (Table 1). Patients completing a telemedicine visit were significantly younger than those completing an in-person office visit (45 years versus 53 years, p = 0.013). There were no significant differences between groups based on sex, race, marital status, religion, insurance, distance to the center, median income, personal history of cancer, or referral reason. Genetic testing was recommended for 220 (93%) patients, with no statistically different rates of genetic testing recommendation between those seen in-person compared to those seen via telemedicine (94.4% in-person versus 92.5% telemedicine, p = 0.58; Table 1). Similarly, there was no statistically significant difference in individuals consenting to genetic testing based on appointment type, with 92.6% of telemedicine patients consenting to genetic testing and 91.7% of in-person office visit patients consenting to genetic testing (p = 0.79). Notably, patients seen via telemedicine had a significantly lower rate of completing genetic testing compared to those seen via an in-person office visit (81.7% vs. 94.8%, p = 0.008). Of the 23 individuals that were seen through telemedicine that consented to testing, but did not have completed testing: 2 (8.7%) had sample failures, 4 (17.4%) shared they were having second thoughts about pursuing testing, and the remaining 17 (73.9%) did not have any discernable reason for not returning a sample. In multivariable analysis, appointment type was the only significantly associated variable, with telemedicine visits associated with statistically significant decreased odds of completing genetic testing after it was recommended (OR 0.26, 95% CI 0.08–0.78, p = 0.02; Supplementary Table 2) relative to in-person office visits. Additionally, there were significant differences in turnaround time for genetic testing results. Patients seen in-person completed testing and received results in a median of 13 days (IQR 9, 18) which was significantly shorter than those seen via telemedicine who completed and received results in a median of 32 days (IQR 22, 56; p < 0.001; Fig. 3).

### Qualitative Survey results

A total of 66 patients completed the verbally administered survey, including 36 (54.5%) who were seen by telemedicine and 30 (45.5%) who were seen in-person (Table 2). Most patients had received a COVID-19 vaccination prior to their appointment (88%) and had previous experience using telemedicine (85%). Patient satisfaction was high for both telemedicine and in-person appointments, with 99% of patients saying their visit met their expectations. Fifty seven (86%) survey participants had a preference for a specific visit type, with most patients (44 participants, 77%) indicating that they preferred a telemedicine appointment, regardless of which modality their visit was actually scheduled as (Table 2). Reasons for preferring telemedicine amongst patients included a reduction in travel time and associated costs such as parking, and less need to secure childcare and take time off from work (Supplementary Table 3). Survey participants also preferred telemedicine because it reduces potential COVID-19 exposures, allows family members to easily join the visits, and lessens the appointment burden for individuals actively undergoing cancer treatment. Reasons why survey participants preferred in-person appointments were that they preferred face-to-face communication with their healthcare provider, there were no technical issues to worry about, and they felt in-person visits were more thorough and made it easier to ask questions (Supplementary Table 3).

**Table 2:** Results of patient surveys administered in GI-CREP clinic

	All Participants (N = 66)	Telemedicine (N = 36)	In-Person Office Visit (N = 30)	p value
Previously tested positive for COVID-19	3 (5%)	1 (3%)	2 (7%)	0.587
Received a COVID-19 vaccine	58 (88%)	33 (92%)	25 (83%)	0.453
Previously used telemedicine	56 (85%)	31 (86%)	25 (83%)	1
Had a preference for either a telemedicine or in-person visit (independent of visit type scheduled)	57 (86%)	32 (89%)	25 (83%)	0.721
Prefer telemedicine (of those with a visit preference)		31 (97%)	13 (52%)	0.0001
Prefer in-person (of those with a visit preference)		1 (3%)	12 (48%)	
Visit met expectations	65 (99%)	36 (100%)	29 (97%)	0.455

## Discussion

The COVID-19 pandemic led to significant changes in healthcare delivery, including the increased use of telemedicine, and understanding the impact of these changes on cancer risk assessment is critical to ensuring the effective delivery of these important services. There have been several studies examining the feasibility and non-inferiority of telemedicine services over time, showing that the use of telemedicine services in many healthcare specialties does not sacrifice quality of care provided [16–18]. However, prior studies before the COVID-19 pandemic have less relevance to our current healthcare landscape post-COVID, as telemedicine has now become a more common and familiar fixture in our healthcare framework. Throughout the pandemic the GI-CREP at our institution utilized both in-person and telemedicine appointments, and we utilized this unique clinic set-up to perform side-by-side comparison of these two different modalities. This study illustrates that although telemedicine was favored by most patients, it did not substantially impact the likelihood of GI-CREP appointment completion, of genetic testing recommendation by providers, or of consent for genetic testing by patients. However, telemedicine use was associated with lower rates of genetic testing completion and a longer turnaround time for receipt of results.

The use of telemedicine can reduce barriers to care for certain patient populations [19]. Patients can avoid traveling long distances, reduce time off work, and importantly, avoid potential COVID-19 exposures. The latter is especially important in the cancer-risk evaluation setting as patients with active cancers may be immunocompromised. In this study’s survey cohort, most patients indicated that they preferred the telemedicine modality with a common reason being that it reduces both the time and costs associated with traveling to the clinic. While telemedicine helps to lower these barriers, it can also introduce new barriers. In order to complete a virtual appointment, the patient must have reliable phone or internet service, and be familiar with the technology used to access their visit. Although it is relatively common for US adults to have access to an internet connection, there are some who do not and therefore may have difficulty accessing a telemedicine appointment. Pew reported in 2021 that approximately 7% of US adults do not have access to the internet, and the National Telecommunications and Information Administration (NTIA) reports that 1 in 5 US households does not have internet access in the home [20, 21]. Telemedicine appointments are also subject to technical glitches such as frozen video or audio cutting out, despite proficiency with technology. In a systematic review article published in 2022, technical issues were found to be the most common issues reported with the use of telehealth [22].

Although telemedicine may lower some barriers, our study found that being scheduled for a telemedicine appointment was not associated with higher rates of appointment completion compared to traditional in-person visits. Possible explanations for why appointment completion rates remain similar for in-person and telemedicine visits are that individuals may be less invested in a telemedicine visit. For example, it may be easier to forget about the appointment if patients do not need to make special arrangements to attend. It may be more difficult for individuals with cancer to attend an extra appointment, regardless of modality, when their schedule can already be full of healthcare visits. Furthermore, technology issues related to telemedicine appointments may prevent patients from logging in to attend their appointments. However, as patients in this cohort were not randomly assigned to a telemedicine or in-person visit type, self-selection bias is certainly possible, which could only be overcome by a future clinical study where individuals are randomized to appointment type.

Overall, we found that 81% of patients completed a GI-CREP appointment, which was higher than completion rates pre-pandemic (75%).^11^ Pre-pandemic we showed that certain factors including Medicaid insurance, self-identified Black race, and a personal history of cancer were associated with lower rates of GI-CREP appointment completion [11]. Fortunately in this study there were no differences in appointment completion rate based on race/ethnicity during the COVID-19 pandemic (Table 1). However, disparities in appointment completion remained based on insurance status and history of cancer, with individuals with Medicaid insurance and a personal history of cancer having lower rates of GI-CREP appointment completion during the COVID-19 pandemic. Given the presence of disparities based on insurance status and personal history of cancer that are observed both pre- and post-pandemic, it is unlikely that pandemic-specific factors were the primary drivers of these disparities.

Our findings demonstrated that the completion rate for genetic testing was significantly different between telemedicine and in-person appointments. In fact, patients seen via telemedicine had a more than three-fold higher chance of not completing genetic testing after consenting compared to patients seen in-person (18.3% of telemedicine patients compared to 5.2% of in-person patients, Table 1). The high rate of genetic testing completion for in-person appointments is likely because patients typically have their blood or saliva sample collected during the in-person appointment. For patients seen via telemedicine, sample collection happens after visit completion, with a saliva kit typically mailed to the patient’s personal address, which could take days or weeks depending on mail delays. During this time, individuals may have second thoughts about following through with their testing, lose interest in the possible results, or become busy and not prioritize the sample collection [23]. Additionally, there could also be increased rates of inadequate or improper sample collection by patients when they are collecting their sample at home without the direct guidance of a genetics professional [24]. Based on the results from this study, new processes were implemented within GI-CREP to improve testing completion rates. Briefly, this process involves requesting monthly reports from commercial laboratories listing patients with outstanding genetic testing samples. Using these lists, a genetic counseling assistant (GCA) will first remind patients about sample collection through the electronic medical record, and if the sample remains outstanding the GCA will attempt to contact the patient by phone. Future studies will be able to assess whether these newly implemented processes improve testing completion rates.

This study also observed significant differences in turnaround time for the return of genetic testing results. Return of results took more than twice as long for telemedicine visits compared to their in-person visit counterparts (32 days for telemedicine compared to 13 days for in-person). It is likely that these delays in return of results are at least partially related to extra time required to mail a sample collection kit to patients and the delay of independent sample collection on the patient’s own accord [24]. The discrepancy in turnaround time between telemedicine and in-person visits is an important issue to consider in the continued use of telemedicine services, especially in the cancer risk assessment setting when genetic testing results could potentially impact treatment options or surgical decision-making.

Limitations of this study include data collection from a single institution GI cancer risk evaluation program. Another limitation is that patients were offered the first available GI-CREP appointment but could choose to wait longer for a different appointment type, and therefore they were not randomly assigned telemedicine or in-person appointments. However, in contrast to cancer genetics programs that transitioned entirely to telemedicine during the COVID-19 pandemic, our clinic structure allowed a head-to-head comparison of these two different appointment modalities within the same time period. Another potential limitation is how translatable our data may be moving forward from the COVID-19 pandemic, as this research was conducted towards the beginning of the pandemic where patients may have had limited experience with telehealth and may have been uncomfortable visiting healthcare facilities in-person.

In conclusion, telemedicine has been a vital service delivery option for GI cancer risk evaluation during the COVID-19 pandemic. When comparing in-person and telemedicine visits, there were no differences in providers recommending genetic testing nor in rate of patient consent for genetic testing. However, more than threefold more patients seen by telemedicine did not complete genetic testing, and return of results took more than twice as long compared to in-person visits. Therefore, it is important to continue to address logistical challenges related to telemedicine including ensuring reliable and expedient sample return through use of increased GCA-driven patient reminders, while simultaneously maintaining the option for in-person visits for patients who are not comfortable using telehealth as well as those where expedited return of results is important for immediate clinical decision-making.

## Electronic supplementary material

Below is the link to the electronic supplementary material.

Supplementary Material 1

## Data Availability

The datasets used and/or analyzed during this study are available from the corresponding author on reasonable request.

## References

[1] Foulkes WD (2008). Inherited susceptibility to common cancers. New England Journal of Medicine.

[2] Lichtenstein P, Holm NV, Verkasalo PK, Iliadou A, Kaprio J, Koskenvuo M (2000). Environmental and heritable factors in the causation of Cancer — analyses of cohorts of twins from Sweden, Denmark, and Finland. New England Journal of Medicine.

[3] Susswein LR, Marshall ML, Nusbaum R, Vogel Postula KJ, Weissman SM, Yackowski L (2016). Pathogenic and likely pathogenic variant prevalence among the first 10,000 patients referred for next-generation cancer panel testing. Genetics in Medicine.

[4] Riley BD, Culver JO, Skrzynia C, Senter LA, Peters JA, Costalas JW (2012). Essential elements of genetic cancer risk assessment, counseling, and testing: updated recommendations of the National Society of genetic counselors. J Genet Couns.

[5] Katabathina VS, Menias CO, Khanna L, Murphy L, Dasyam AK, Lubner MG (2019). Hereditary Gastrointestinal Cancer Syndromes: role of imaging in screening, diagnosis, and management. RadioGraphics.

[6] Hampel H (2016). Genetic counseling and cascade genetic testing in Lynch syndrome. Fam Cancer.

[7] Beatrice Godard AP, Martine, Dumont (2007). Adele Simard-Lebrun, Jacques Simard factors Associated with an Individual’s decision to withdraw from genetic testing for breast and ovarian Cancer susceptibility: implications for Counseling. Genetic Testing.

[8] Olusola P, Banerjee HN, Philley JV, Dasgupta S (2019). Human Papilloma Virus-Associated Cervical Cancer and Health Disparities. Cells.

[9] Yedjou CG, Sims JN, Miele L, Noubissi F, Lowe L, Fonseca DD (2019). Health and racial disparity in breast Cancer. Adv Exp Med Biol.

[10] Singh GK, Jemal A (2017). Socioeconomic and Racial/Ethnic disparities in Cancer Mortality, incidence, and Survival in the United States, 1950–2014: over six decades of changing patterns and widening inequalities. J Environ Public Health.

[11] Ebrahimzadeh JE, Long JM, Wang L, Nathanson JT, Siddique SM, Rustgi AK (2020). Associations of sociodemographic and clinical factors with gastrointestinal cancer risk assessment appointment completion. J Genet Couns.

[12] Mann DM, Chen J, Chunara R, Testa PA, Nov O (2020). COVID-19 transforms health care through telemedicine: evidence from the field. Journal of the American Medical Informatics Association.

[13] Gajarawala SN, Pelkowski JN (2021). Telehealth benefits and barriers. J Nurse Pract.

[14] Kruse C, Fohn J, Wilson N, Nunez Patlan E, Zipp S, Mileski M (2020). Utilization barriers and medical outcomes commensurate with the Use of Telehealth among older adults: systematic review. JMIR Med Inform.

[15] H.R. 1892 Bipartisan Budget Act of 2018, (2018).

[16] Kummervold PE, Johnsen J-AK, Skrøvseth SO, Wynn R (2012). Using noninferiority tests to evaluate telemedicine and e-health services: systematic review. Journal of medical Internet research.

[17] Bradbury A, Patrick-Miller L, Harris D, Stevens E, Egleston B, Smith K (2016). Utilizing Remote Real-Time videoconferencing to Expand Access to Cancer Genetic Services in Community Practices: a Multicenter Feasibility Study. J Med Internet Res.

[18] Cacioppo CN, Egleston BL, Fetzer D, Burke Sands C, Raza SA, Reddy Malleda N (2021). Randomized study of remote telehealth genetic services versus usual care in oncology practices without genetic counselors. Cancer Med.

[19] Bressman E, Werner RM, Childs C, Albrecht A, Myers JS, Adusumalli S. Association of Telemedicine with Primary Care Appointment Access after Hospital Discharge. J Gen Intern Med. 2022:1–3. 10.1007/s11606-021-07321-3 PMC8751457 35018569

[20] Perrin A, Atske S. 7% of Americans don’t use the internet. Who are they? Pp. Pew Research center, pewresearch.org. https://www.pewresearch.org/fact-tank/2021/04/02/7-of-americans-dont-use-the-internet-who-are-they/

[21] National Telecommunications and Information Administration (NTIA). Switched off: why are one in five U.S. households not online? Pp. United States Department of Commerce, ntia.gov. https://ntia.gov/blog/2022/switched-why-are-one-five-us-households-not-online#:~:text=Internet%20access%20means%20access%20to,to%20the%20Internet%20at%20home.

[22] Ftouni R, AlJardali B, Hamdanieh M, Ftouni L, Salem N. Challenges of Telemedicine during the COVID-19 pandemic: a systematic review. BMC Med Inform Decis Mak. 2022;22(1):207. Published 2022 Aug 3. doi:10.1186/s12911-022-01952-0 10.1186/s12911-022-01952-0 PMC9351100 35922817

[23] Uhlmann WR, McKeon AJ, Wang C (2021). Genetic counseling, virtual visits, and equity in the era of COVID-19 and beyond. J Genet Couns.

[24] Ahimaz P, Giordano J, Disco M, Harrington E, Levinson E, Spiegel E (2021). COVID contingencies: early epicenter experiences of different genetics clinics at a New York City institution inform emergency adaptation strategies. J Genet Couns.

