# Supplementary material for: Comparing telemedicine and in-person gastrointestinal cancer genetic appointment outcomes during the COVID-19 pandemic
Source: Hered Cancer Clin Pract. 2023 May 8;21:6. doi: 10.1186/s13053-023-00250-8 (PMC10165576; doi:10.1186/s13053-023-00250-8)
Supplement: Supplementary file 1 — Supplementary Material 1 [file 13053_2023_250_MOESM1_ESM.docx]

**SUPPLEMENTARY DATA**

**Supplementary Table 1 – Univariable and Multivariable Logistic Regression Models for Appointment Completion**

|  | **Univariable Analyses** | | **Multivariable Model** | |
| --- | --- | --- | --- | --- |
| **Variable** | **Odds Ratio (95% CI)** | **p-value** | **Odds Ratio (95% CI)** | **p-value** |
| **Referral Reason** |  |  |  |  |
| Family history of cancer | (ref) |  | (ref) |  |
| Personal history of cancer | 0.28 (0.13-0.60) | 0.001 | 0.27 (0.12-0.62) | 0.002 |
| Personal history of polyps | 2.76 (0.58-13.15) | 0.20 | 2.97 (0.60-14.68) | 0.18 |
| Family history of genetic syndrome | 1.00 (0.20-4.98) | 1.00 | 0.85 (0.17-4.35) | 0.85 |
| Other | 0.09 (0.03-0.30) | <0.001 | 0.11 (0.03-0.37) | <0.001 |
| **Insurance** |  |  |  |  |
| Private | (ref) |  | (ref) |  |
| Medicare | 0.50 (0.25-1.02) | 0.06 | 0.61 (0.29-1.29) | 0.20 |
| Medicaid | 0.32 (0.13-0.78) | 0.01 | 0.30 (0.11-0.82) | 0.02 |
| Other/Unknown | 0.04 (0.00-0.39) | 0.005 | 0.05 (0.00-0.48) | 0.01 |
| **Age (per 1 year)** | 0.98 (0.96-1.00) | 0.07 | - | - |

** Appointment type (telemedicine vs. office visit) was not significantly associated with the outcome*

**Supplementary Table 2 – Univariable and Multivariable Logistic Regression Models for Completion of Genetic Testing (among those where it was recommended)**

|  | **Univariable Analyses** | | **Multivariable Model** | |
| --- | --- | --- | --- | --- |
| **Variable** | **Odds Ratio (95% CI)** | **p-value** | **Odds Ratio (95% CI)** | **p-value** |
| **Visit Type** |  |  |  |  |
| In-Person (reference) | (ref) |  | (ref) |  |
| Telemedicine | 0.28 (0.09-0.85) | 0.03 | 0.26 (0.08-0.78) | 0.02 |
| **Insurance** |  |  |  |  |
| Private | (ref) |  |  |  |
| Medicare | 2.32 (0.51-10.61) | 0.28 | - | - |
| Medicaid | 0.33 (0.10-1.07) | 0.07 | - | - |
| Other/Unknown | - | - | - | - |

**Excludes one patient who died shortly after consenting to genetic testing*

**Supplementary Table 3 - Reasons for telemedicine versus in-person appointment preference provided by survey respondents.**

| **Reasons patients prefer telemedicine visits (N=44)** | **Reasons patients prefer in-person visits (N=13)** |
| --- | --- |
| Saves on travel time and costs | Prefer face-to-face communication with a provider |
| Do not need to secure childcare during these visits | Feel that the first visit should be in-person to establish a doctor-patient relationship |
| Easier appointments, especially if physical exam is not required | In-person visits are more thorough, and it is easier to ask more questions |
| Avoids COVID exposures | Offers privacy away from family members |
| Do not need to take time off from work | No technical issues to worry about |
| Family members can easily join the visit | Clinic is close to home/work so it is convenient to come in-person |
| Lessens appointment burden for patients currently undergoing cancer treatment |  |
| More personable to see a provider’s face without a mask on |  |
